# Evaluating the Mechanical Properties of Restorative Glass Ionomers Cements

**DOI:** 10.1016/j.identj.2022.06.016

**Published:** 2022-08-26

**Authors:** Sarah Bahammam, Dan Nathanson, Yuwei Fan

**Affiliations:** a Department of Pediatric Dentistry and Orthodontics, College of Dentistry, Taibah University, Medina, Kingdom of Saudi Arabia; b Department of Restorative Sciences and Biomaterials, Boston University Henry M. Goldman School of Dental Medicine, Boston, Massachusetts, USA

**Keywords:** Compressive strength, Glass ionomer cement, Mechanical properties, Restoration, Retention power

## Abstract

**Objective:**

The aim of this research was to assess the efficiency of 4 restorative glass ionomer cements (GICs): Fuji IX (GC), ChemFil Rock (DENSPLY), Riva Self-Cure (SDI), and Ketac Nano (3M ESPE).

**Materials and methods:**

The 4 restorative glass ionomers’ diametral tensile and compressive strengths were evaluated at room temperature for 24 hours and then stored in distilled water. The universal testing machine (INSTRON 5566A) was used to record the maximum load necessary to fracture specimens. Surface wear, diametral tensile strength, and compressive strength against dental ceramic were compared using analysis of variance followed by the Bonferroni method at a significance level of 0.05.

**Results:**

Ketac Nano and ChemFil Rock were found to have better diametral tensile strength than Riva Self-Cure and Fuji IX. The significant difference between ChemFil Rock and Fuji IX (*P* ≤ .005) and ChemFil Rock with Riva Self-Cure (*P* ≤ .005) was shown by post hoc analysis. Ketac Nano had better tensile strength than Riva Self-Cure and Fuji IX. Fuji IX showed the lowest material loss of the GICs as revealed by wear against VITABLOCS Mark II (VITA Zahnfabrik).

**Conclusions:**

This study indicated a significant difference in the compressive strengths of ChemFil Rock and Riva Self-Cure. ChemFil Rock had the highest tensile strength. The diameter tensile strength of all 4 materials was statistically insignificant. Finally, Fuji IX had the least amount of material loss. ChemFil Rock was proven to be more effective than Fuji IX.

## Introduction

Recent advancement in dentistry compels the dominating use of dental restorative materials in the market. Ceramic and gold as indirect restorative materials are common, whilst amalgam has been utilised in direct restorations.^1^ Amalgam use has been restricted due to allergy and toxic reactions.^2^ A variety substitutes (resin composites and glass ionomer cements [GICs]) have evolved as a result of significant advances in dental research.^3^ The most aesthetically pleasing materials with acceptable physical qualities are resin composites.^4^ They have disadvantages in that they are a very costly, time-consuming, and technique-dependent adhesive treatment.^5^ Biocompatibility, chemical union to mineralised tissue, and continual fluoride release are only a few of the benefits of using these materials.^6^ Inferior mechanical qualities, such as reduced fracture strength, toughness, and wear, preclude their usage in dentistry.^7^^,^^8^ GICs are often used as a material for temporary fillings in the posterior dental area.^9^

These characteristics are especially significant when using orthodontic bands to prevent the occurrence of caries and periodontal disorders whilst also providing enough union strength between teeth and bands.^10^^,^^11^ The study by Jayanthi and Vinod^12^ investigated flexural strength and compressive strength. However, no previous study has collectively evaluated diametral tensile strength, compressive strength, material loss, and wear resistance. The cost of GICs is considered in the majority of public and private health care setups by health care professionals, and it influences the selection of GICs. Investigating the compressive strength of ionomers is imperative, as it helps in understanding the mechanical integrity of a specific material. The present study aimed to compare the mechanical characteristics of 4 restorative GICs: ChemFil Rock (DENSPLY), Fuji IX (GC), Ketac Nano (3M ESPE), and Riva Self-Cure (SDI).

## Materials and methods

### Specimen synthesis

ChemFil Rock, Fuji IX, Ketac Nano, and Riva Self-Cure were the 4 restorative glass ionomers tested in this study, which used an in vitro experimental study design. These materials’ diametral tensile and compressive strengths were evaluated at room temperature for 24 hours and then stored in distilled water. In a Teflon mould with a 6-mm depth, glass ionomer specimens measuring 6 mm in diameter and 3 mm in height were created. After that, the components were sandwiched between 2 tiny glass slides. A single operator followed the manufacturer's instructions for the synthesis of specimens. A digital calliper was used to assess the accuracy of specimens before beginning the experimental method. All of the specimens were light cured using Coltolux 75 (Coltène/Whaledent). After polymerisation, the research samples were kept at 100% relative humidity. During cross-sectioning on silicon carbide sheets, wet grinding (Mecapol P 251, PRESI) was performed, followed by slurry polishing. All study specimens were ultrasonically cleaned through Axtor CD–4820 after placing them for 15 minutes in distilled water, before further continuing the procedure.

### Mechanical testing and microstructure analyses

**Fig. 1:**
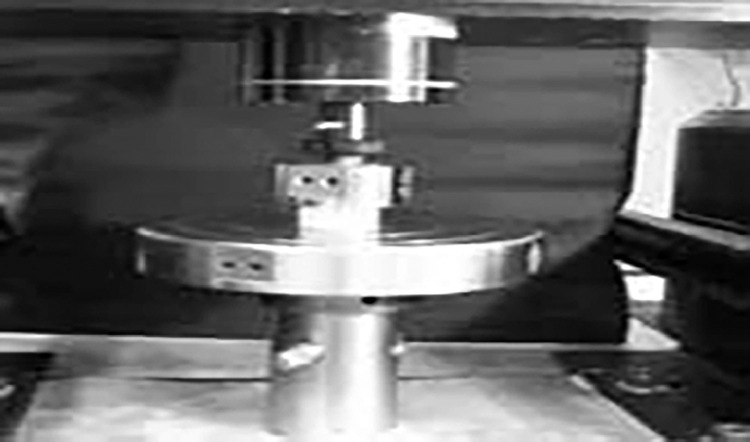
Universal testing machine (INSTRON 5566A).

The compressive and diametral tensile strengths of materials were calculated. The universal testing machine (INSTRON 5566A) was used to record the maximum load necessary to fracture specimens (Figure 1). For each of the GICs, 20 specimens were prepared for a duration of 1 hour to determine diametral tensile strength (DTS), compressive strength (CS), and wear time. The CS calculation was done using the equation CS = 4*P*/π*d*2, where *P* = maximum applied force a at fracture and *d* = specimen diameter.^13^ The DTS was determined from the relationship DTS = 2*P*/πDT, where *P* is load at fracture and *D* = diameter and *T* = specimens thickness.^14^ In the compressive strength test, specimens were loaded with flat ends between the platens of the apparatus; consequently, the load may be applied to the long axis at a crosshead speed of 0.5 mm/min, which is the same as the crosshead speed employed by Bhattacharya et al^15^ and Sulaiman et al.^16^ For diametral tensile strength testing, the specimens were placed with flat ends perpendicular to the platens of the apparatus, and the load was applied to the diameter of the specimens at a crosshead speed of 0.5 mm/min. The Leica Application Suite software (Leica Microsystems) was used to link the microscope to the computer, and the black-and-white pictures were analysed using Adobe Photoshop (Adobe Systems Software).

### Wear tests

The specimens were kept in distilled water for 24 hours before commencing the wear testing. In vitro wear against VITABLOCS Mark II (VITA Zahnfabrik) was measured using the standard flat pin-on-plate method. In this 2-body abrasion test, no abrasive substance was employed. The experiment was set up by inserting the plates in a rectangular mould with dimensions of 10 mm long, 3 mm wide, and 2 mm deep. The materials to be tested were put into the mould and allowed to cure for the necessary amount of time before being immersed in water for 100,000 abrasive cycles (30 cycles/min) of linear wear. VITABLOCS Mark II was used to make antagonist pins with a diameter of 2.6 mm. With a lateral movement of 5 mm powered by a rotary motor, consistent contact between opposing pins and the testing material surface was maintained at a weight of 90 gm. Using an optical profilometer, the depth of the groove generated in micrometres (m) was measured (Mahr Perthen). Selected glass ionomer specimens were examined using a scanning electron microscope with a backscattering electron detector to perform a comparison between the abrasion part and the undamaged section. The specimens were sputter-coated with palladium and gold using a sputter coater (Hummer II Technics) (25% Pd/75% Au). The wear of the glass ionomer surface induced by the ceramic VITABLOCS Mark II cylinders was examined using a depth micro analyser.

### Data collection and analysis

Surface wear, diametral tensile strength, and compressive strength against dental ceramic were compared using analysis of variance followed by the Bonferroni method at a significance level of 0.05. When numerous pairwise evaluations are performed with a single data set,^17^ the Bonferroni method is used to compute the correlation, which is utilised to reduce the appearance of false-positive results and detect significant differences in the materials in each group.

## Results

**Fig. 2:**
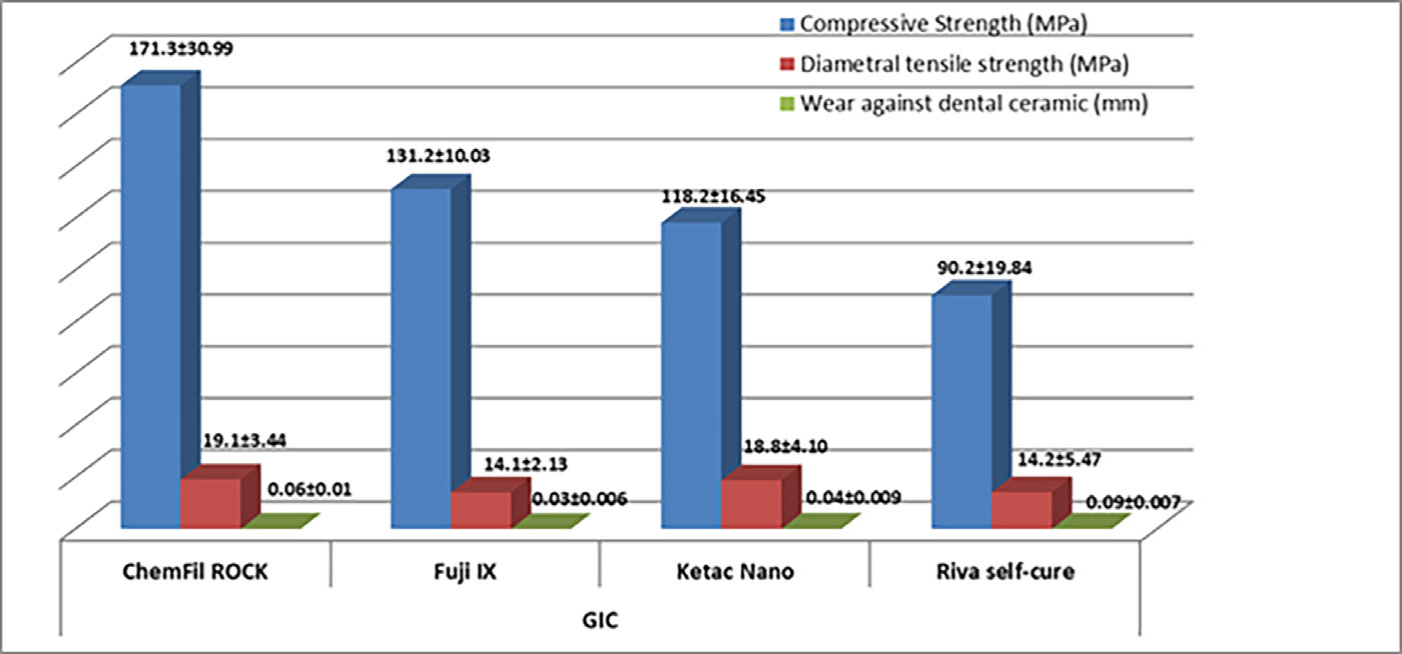
Mean diametral tensile strength, compressive strength, and material loss of glass ionomer cements.

**Fig. 3:**
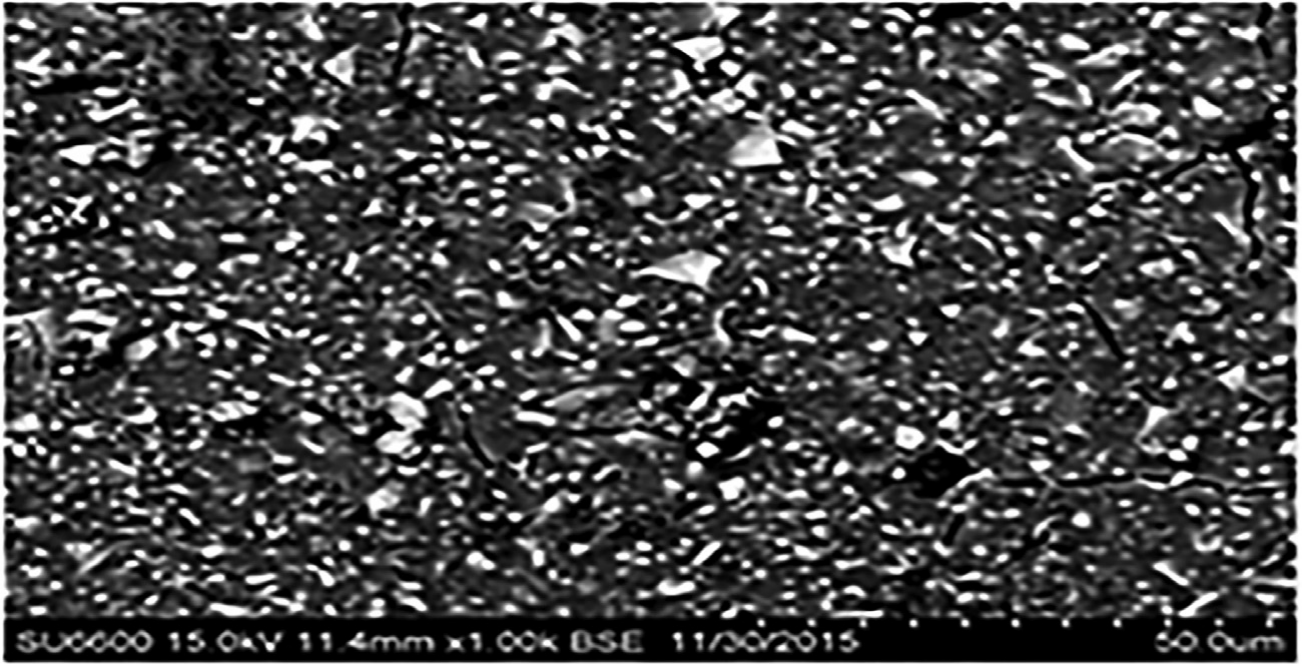
ChemFil Rock (intact area) at a 1000× magnification.

**Fig. 4:**
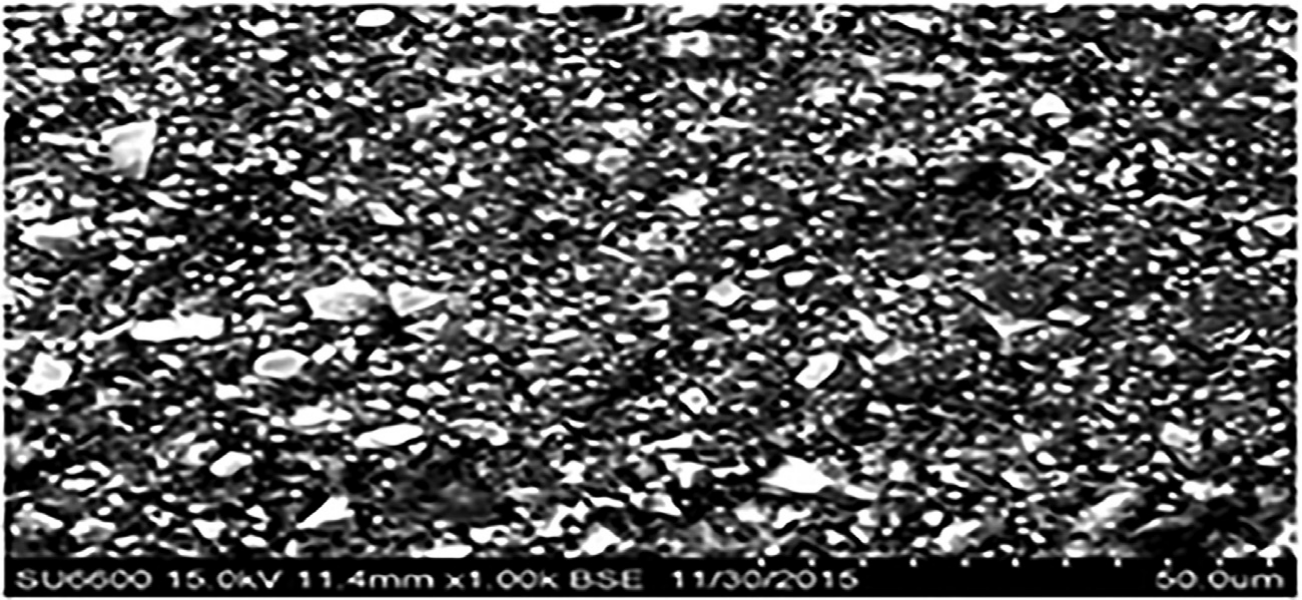
ChemFil Rock (abrasion area) at 1000× magnification.

**Fig. 5:**
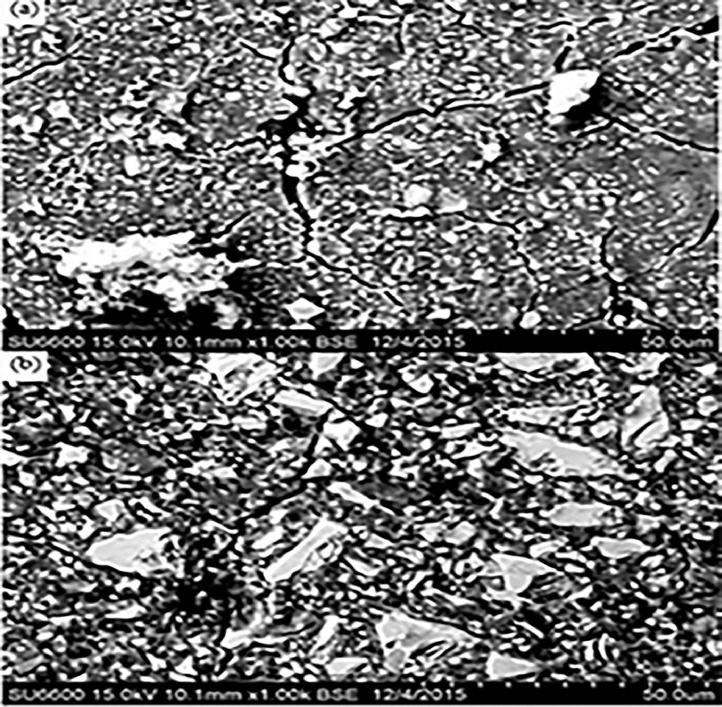
Riva Self-Cure: A, intact; B, abrasion area at 1000× magnification.

**Fig. 6:**
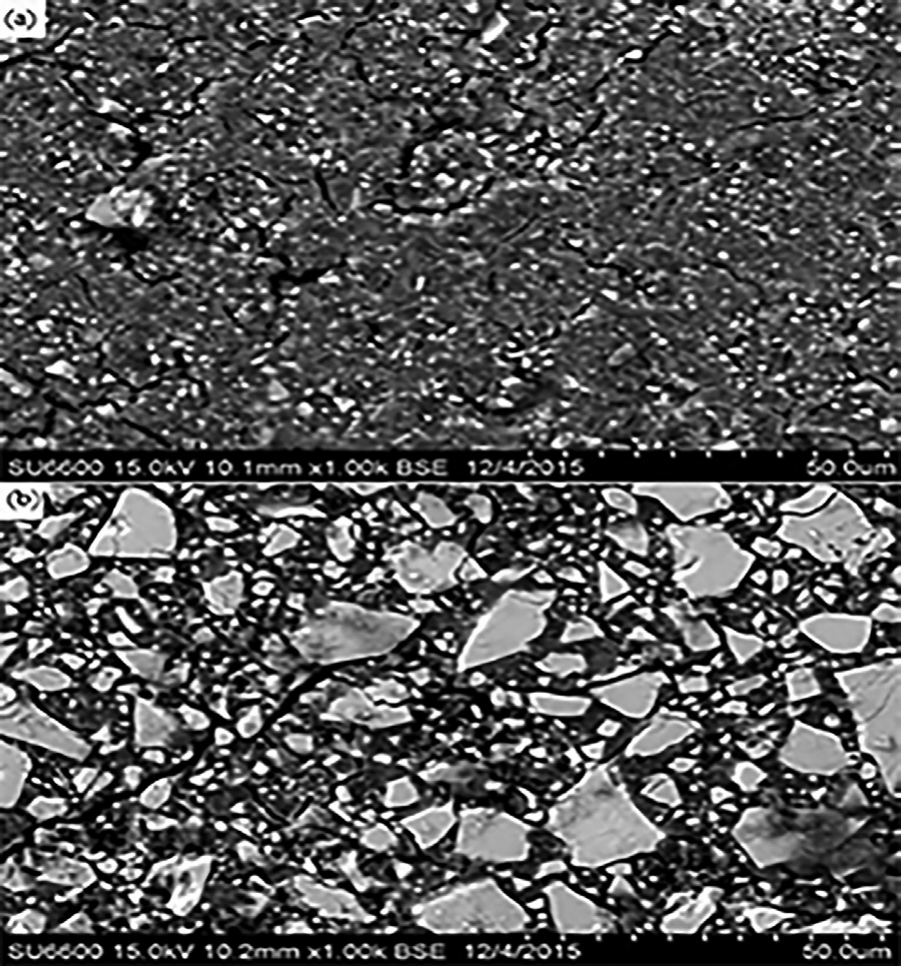
Fuji IX: A, intact; B, abrasion area at 1000× magnification.

**Fig. 7:**
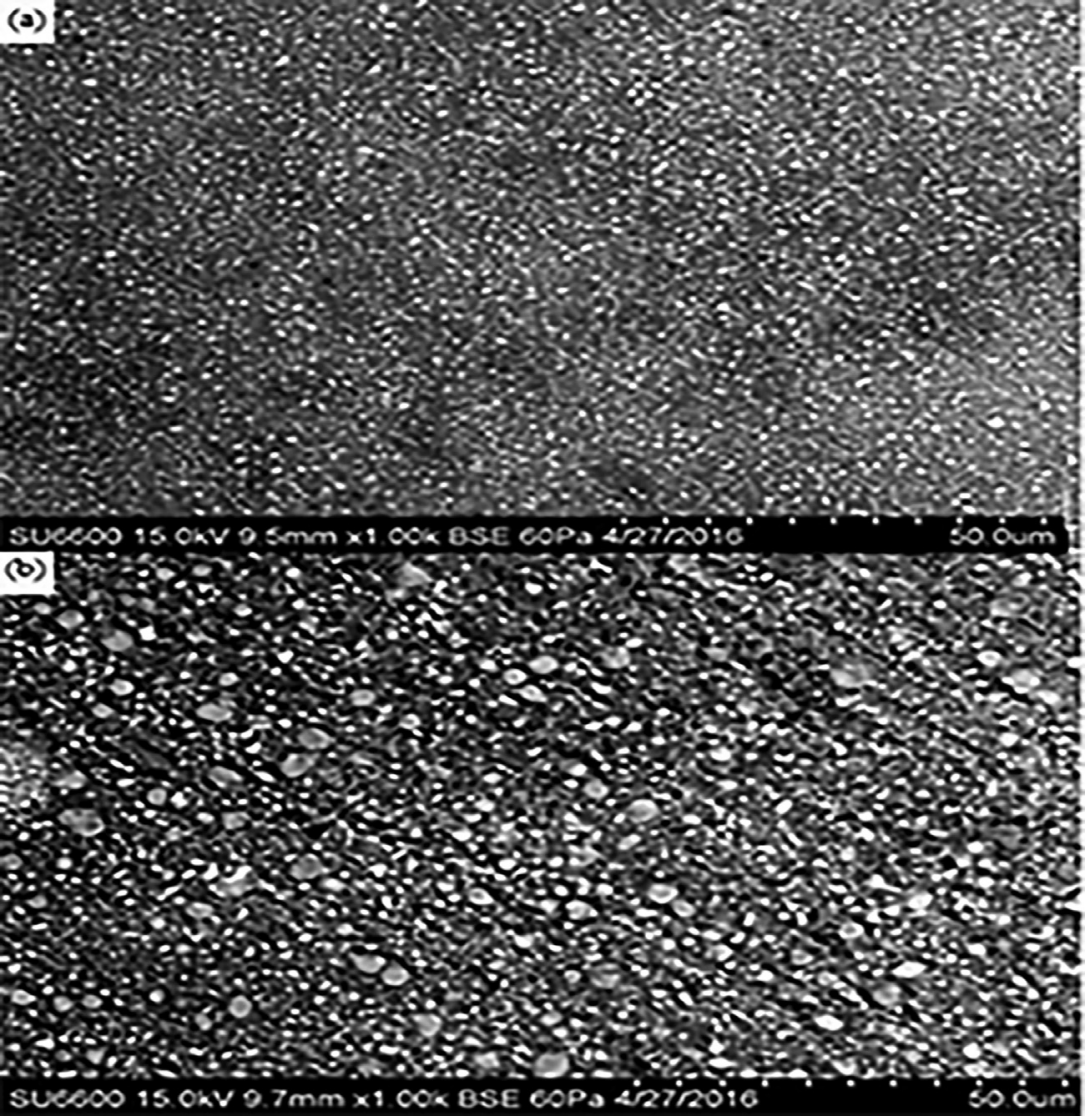
Ketac Nano: A, intact; B, abrasion area at 1000× magnification.

The wear resistance, compressive strength, and diametral tensile strength of the 4 restorative glass ionomers were investigated. Chemical composition of glass ionomers is shown in Table 1. Figure 1 shows the mean material loss, diametral tensile strength, and compressive strength. ChemFil Rock (171.3 ± 30.99 MPa) was found to have highest mean compressive strength followed by Fuji IX (131.2 ± 10.03 MPa) and Ketac Nano (118.2 ± 16.45 MPa). ChemFil Rock (19.1 ± 3.44 MPa) was found to have better diametral tensile strength than Riva Self-Cure (14.2 ± 5.47 MPa) and Fuji IX (14.1 ± 2.13 MPa). The significant difference between ChemFil Rock and Fuji IX (*P* ≤ .05) and ChemFil Rock with Riva Self-Cure (*P* ≤ .05) was shown by post hoc analysis. Ketac Nano had better tensile strength (18.8 ± 4.10 MPa) than Riva Self-Cure (14.2 ± 5.47 MPa, *P* ≤ .05) and Fuji IX (14.1 ± 2.13 MPa, *P* ≤ .05). Fuji IX showed the lowest material loss (0.03 ± 0.006 MPa; Table 2). When compared to the fillers in the intact area, the fillers in the abrasion area were more defined, with sharp edges. Furthermore, in the abrasion area, the fillers were more exposed to the surface. The fillers were primarily embedded in the intact area for resin-modified glass ionomers, resulting in a polymer-rich unpolished glass ionomer surface (Fig. 2, Fig. 3, Fig. 4, Fig. 5, Fig. 6, Fig. 7).

**Table 1:** Chemical composition of glass ionomers.

Materials	Composition	Manufacturer
ChemFil Rock (Dentsply)	Polyacrylic and tartaric acid, fluorosilicate, and BHT	VOCO AC, Guxhaven, Germany
Fuji IX (GC)	Fluoroaluminosilicate glass, multifunctional methacrylate, water, methylmethacrylate, polyacrylic acid, camphorquin	GC Corp, Tokyo, Japan
Ketac Nano(3M ESPE)	Al-Ca-La fluorosilicate glass, ethanediyl ester, copolymer acrylic, maleic acid, and aminoethyl ester dicyclopentyldimethylene diacrylate	3M ESPE AG, Seefeld, Germany
Riva Self-Cure (SDI)	Fluoroaluminosilicate glass, acrylic monomer, and polyacrylic acid + tartaric acid	SDI, Victoria, Australia

**Table 2:** Post hoc analysis for the comparison of difference in mean diametral tensile strength, compressive strength, and material loss of GICs.

GIC	Comparison GIC	Compressive strength *P* value	Diametral tensile strength *P* value	Wear against dental ceramic *P* value
ChemFil Rock	Fuji IX	>.05	<.05	<.05
	Ketac Nano	<.05	>.05	<.05
	Riva Self-Cure	<.05	<.05	<.05
Fuji IX	Ketac Nano	>.05	<.05	<.05
	Riva Self-Cure	<.05	>.05	<.05
Ketac Nano	Riva Self-Cure	<.05	<.05	<.05

GIC, glass ionomer cement.

## Discussion

The goal of this in vitro study was to examine the efficiency of 4 different restoration glass ionomers in terms of diametral tensile strength, compressive strength, and material loss. Ketac Nano and ChemFil Rock had greater tensile strength than the others. The tensile strengths of Riva Self-Cure and Fuji IX are statistically different at 14.2 ±5 .47 MPa and 14.1 ± 2.13 MPa, respectively. These results are comparable to those of a study by Pilo et al,^18^ who found the same shear punch strength of glass ionomer cement when coated with varnishes. It was also proposed that the glass ionomer surface's strength may be improved by coating it to prevent it against water-based contamination. ChemFil Rock may be used to include fillers since it produces incremental itaconic acid, which boosts the glass ionomer's resistance capabilities.^19^ The diametral strength of ChemFil Rock and Ketac Nano was higher than that of Fuji IX. Khairina et al^20^ made an interesting point, claiming that the diametral tensile strength of bulk-fill composite resin increases as the storage temperature rises. The approach used in this study allowed for a comparison of varied behaviours of restorative materials, as material loss in all of the materials studied ranged from 0.038 to 0.079 m.

In comparison to the other materials examined, Fuji IX showed the least wear. These findings are consistent with those of Kunzelmann et al,^21^ who discovered that Fuji IX had the least amount of material loss when compared to Ketac Silver and Ketac Molar. Ryu et al^22^ looked at the wear resistance of several GICs and found a substantial difference in early wear rates as well as a considerable decrease in long-term wear rates. Within the time frame of 4 months to 1 year, however, evidence of wear reduction was also detected. Wear resistance tests have indicated that different powder particles impact the interfacial interaction between the polymer matrix and the particles.

According to the findings, ChemFil Rock had the highest compressive strength. The compressive strength of Ketac Molar and Fuji IX was estimated and compared to previously published results.^23^ Research by Zoergiebel et al^24^ looked at the chemical composition of acrylic liquid and filler in 4 different GICs: Fuji IX Fast (GC), Riva Self-Cure (SDI), ChemFil Rock (Dentsply, York, PA, USA), and Fuji IX GP Extra/Equia (GC). Zinc is a prominent component of ChemFil Rock's glass composition. The unique zinc accretion, which is expected to improve reactivity, and the contribution of zinc oxide as a network modifier to the Si–O–Si bond breakage in the glass,^24^ which increases the glass's sensitivity to acid attack,^25^, ^26^, ^27^ imply that this GIC has better mechanical characteristics.

The characteristics of GICs are examined for 24 hours or more after mixing, according to the majority of research.^18^^,^^28^ There are certain limitations to the current study. One of the study's significant flaws is that it only employed 4 restorative materials and did not assess the diametral tensile and compressive strength of each material over time. Furthermore, the test only lasted 24 hours, and no additional evaluations were done based on the time interval. A weakness of the study is that it only considers the initial cost; however, long-term cost effectiveness is unclear. Long-term effect should be tested, however, it was not the scope of this study.

## Conclusions

This study indicated the significant difference in the compressive strengths of ChemFil Rock and Riva Self-Cure. ChemFil Rock had the highest tensile strength. The diameter tensile strength of all 4 materials was statistically insignificant. Finally, Fuji IX had the least amount of material loss. ChemFil Rock was proven to be more effective than Fuji IX. In view of the findings of this study and their comparison to past research, it is recommended that more research be done on the kinetics of GICs and their relationship to various mechanical characteristics on the first day of mixing.

## Conflict of interest

None disclosed.
